# Structure of the stationary phase survival protein YuiC from *B.subtilis*

**DOI:** 10.1186/s12900-015-0039-z

**Published:** 2015-07-11

**Authors:** Doris H.X. Quay, Ambrose R. Cole, Adam Cryar, Konstantinos Thalassinos, Mark A. Williams, Sanjib Bhakta, Nicholas H. Keep

**Affiliations:** Institute for Structural and Molecular Biology, Crystallography, Department of Biological Sciences, Birkbeck University of London, Malet Street, London, WC1E 7HX UK; Institute of Structural and Molecular Biology, Division of Biosciences, University College London, Gower Street, London, WC1E 6BT UK

**Keywords:** Dormancy, Stationary phase survival, Peptidoglycan, Resuscitation promoting factors, Firmicutes

## Abstract

**Background:**

Stationary phase survival proteins (Sps) were found in Firmicutes as having analogous domain compositions, and in some cases genome context, as the resuscitation promoting factors of Actinobacteria, but with a different putative peptidoglycan cleaving domain.

**Results:**

The first structure of a Firmicute Sps protein YuiC from *B. subtilis*, is found to be a stripped down version of the cell-wall peptidoglycan hydrolase MltA. The YuiC structures are of a domain swapped dimer, although some monomer is also found in solution. The protein crystallised in the presence of pentasaccharide shows a 1,6-anhydrodisaccharide sugar product, indicating that YuiC cleaves the sugar backbone to form an anhydro product at least on lengthy incubation during crystallisation.

**Conclusions:**

The structural simplification of MltA in Sps proteins is analogous to that of the resuscitation promoting factor domains of Actinobacteria, which are stripped down versions of lysozyme and soluble lytic transglycosylase proteins.

**Electronic supplementary material:**

The online version of this article (doi:10.1186/s12900-015-0039-z) contains supplementary material, which is available to authorized users.

## Background

Firmicutes, and in particular *Bacillus subtilis,* form spores, through a complex and well-studied differentiation to a very stable endospore, where they can survive for decades until revived; for a recent review see [1]. Firmicutes include the genera *Bacillus* and *Clostridia*, which include the pathogenic agents of anthrax, tetanus and botulism, as well as one of the best-studied laboratory bacteria *B. subtilis*. In contrast in Actinobacteria the dormant state is the most stable form, where the bacteria enter a state of very low metabolic turnover and no cell division from which they are hard to revive [2]. The Actinobacteria *Mycobacterium tuberculosis* (*Mtb*) survives in granulomas in the lung for decades in the dormant state. This can be recapitulated *in vitro* by extended growth of stationary cultures or by hypoxia [3]. The physiological situations where Firmicutes enter and exit a dormant state, or at least a late stationary phase state rather than undergo sporulation, are less well understood in terms of the pathogenic lifecycle than dormancy in Actinobacteria. However, a dormant state in Firmicutes can be recapitulated in the laboratory [4, 5]. This paper describes the first structure of a group of peptidoglycan cleaving enzymes from Firmicutes, called stationary phase survival proteins (Sps), that are involved in survival in the stationary phase and are analogous to the resuscitation promoting factors (Rpfs) from Actinobacteria.

Resucitation promoting factors revive Actinobacteria from dormancy [6]. They have a conserved catalytic domain that cleaves peptidoglycan [7, 8] and structurally resembles a stripped-down version of lysozyme and lytic transglycosylases [7, 9]. They vary in the ancillary domains attached to the catalytic domain. Two models have been proposed for the action of Rpfs, either they release a peptidoglycan fragment that acts as a signal for a receptor or the hydrolysis of the peptidoglycan leads to a physical removal of a block to cell division [2, 10–12]. Recent evidence supports the peptidoglycan fragment model [13].

Stationary Phase Survival (Sps) proteins were discovered by comparison of the domain structure of *M.tuberculosis* RpfB with proteins from bacteria in the Firmicute phylum. This search found a protein, YabE in *B. subtilis*, which had the same ancillary domains (DUF348 × 5-G5-hydrolase) [14]. This led to the proposal of a non-orthologous domain displacement event where the overall ancillary domain architecture of RpfB, and the homology of surrounding genes, was maintained but the Rpf catalytic domain was replaced by another domain widely found in Firmicutes, which was named ‘stationary phase survival’ (Sps) domain [14]. The Sps domain has low, but significant, sequence similarity to the C-terminal region of MltA (membrane-bound lytic transglycosylase A), which contains the 3D (three aspartate) domain motif, that includes the catalytic aspartate, and is consequently a putative peptidoglycan hydrolase of the MltA/3D family [14].

There are five major groups of Sps proteins in Firmicutes based on their domain structure [14]. The SpsA group, which includes the *B.subtilis* protein YocH, contains two LysM domains as well as the Sps domain. LysM domains bind peptidoglycan and are found in a range of proteins including some Rpf proteins, amongst which is the first Rpf to be discovered from *M.luteus* [6]. SpsB group are the proteins that resemble RpfB in ancillary domain structure. YuiC is a member of the SpsC group which, like RpfC [15], RpfD and RpfE, only has a signal peptide and short extensions beyond the conserved catalytic domain. The SpsD group contains a COG3883 domain, only found in putative peptidoglycan cleaving enzymes in Firmicutes, in addition to the catalytic domain. There is no example of this group in *B.subtilis,* nor of the SpsE group that contain two SH3b domains, again found in peptidoglycan cleaving enzymes, as well as the catalytic domain. *B.subtilis* does have a fourth Sps protein YorM located on a phage in the chromosome, which are classed in a minor group [14].

Ravagnani *et al.* [14] proposed that the Sps proteins were not involved in spore formation and germination, but in the prolonged survival of these bacteria in stationary phase prior to spore formation. Studies have looked at the effect of deleting the archetypal Sps from *B. subtilis*, YocH [4], and deleting both Sps proteins in *Listeria monocytogenes* [16]. Shah and Dworkin [4] showed the YocH was induced by muropeptides, *via* the Ser/Thr kinase PrkC, was active in degrading peptidoglycan in a zymogram and that deletion of YocH compromised survival in stationary phase, which could be rescued by other bacteria secreting YocH. However, YocH is only one of three genomic Sps proteins in *B. subtilis* (with a further Sps protein on a phage carried in many strains). Pinto *et al.* [17] showed that knockouts of the Sps proteins in *L. monocytogenes* extend the lag phase for growth on minimal medium, but have no effect on rate or duration of growth in the exponential phase. Recently a dormant state was shown in the pathogenic Firmicute*, Staphylococcus aureus,* that could be revived by culture supernatant, analogous to the behaviour of Actinobacteria*.* However, the paper did not directly show that an Sps protein was the active factor [5].

In this paper we present three high-resolution crystal structures of YuiC, an Sps protein from *B. subtilis*. These are of the apo-enzyme (Apo), of the enzyme with a monosaccharide, *N*-acetylglucosamine (NAG) bound in part of the active site (+NAG) and with a 1,6-anhydro-*N*-acetylglucosmaine-*N*-acetylglucosmaine disaccharide product (+Anhydro) arising from incubation of the enzyme with penta-*N*-acetyl-penta-glucosamine (penta-NAG). This defines for the first time the boundaries of the Sps domain and confirms that it is a minimal catalytically active version of the MltA structure. This structural simplicity is analogous to that of the Rpf domain being a minimal version of the Slt/lysozyme fold.

## Results and discussion

### Protein expression and structure solution

*B. subtilis* YuiC 32–218 (Uniprot J7JYQ4_BACIU), which lacks only the predicted signal peptide, was purified after cytosolic expression in *E. coli*. This protein ran as two peaks on gel filtration corresponding to probable monomer and dimer fractions based on the elution volume (Additional file 1: Figure S1). The sample also showed partial proteolysis to give a lower molecular weight band on SDS-PAGE (Additional file 1: Figure S2). Peptide mapping by mass spectrometry indicated that this was likely to be cleavage at or close to R52 based on the sequence found in the Uniprot database. NMR spectroscopy of the truncated fragment indicated that there was loss of a series of sharp peaks in the amide region compared to a full-length sample, consistent with loss of an unstructured region at the N-terminus (Additional file 1: Figure S3). Subsequent N-terminal truncated constructs (P73-E218 and P73-K217), designed to remove the disordered regions of the protein, still ran with two peaks on gel filtration. So far only the first-eluting gel filtration (dimer) peak ever produced crystals of any construct.

Three structures of YuiC have been solved by molecular replacement based on distant homology to *E. coli* MltA (see Experimental Procedures). Two are in space group R3 with two chains forming a tight dimer in the asymmetric unit. Both structures contain ligands bound to both chains. One contains the partial substrate N-acetylglucosamine (+NAG) (PDB 4WJT). The other (+anhydro) was grown in the presence of penta-NAG (PDB 4WLK), but with clear density in each chain for a 1,6-anhydro-*N*-acetylglucosamine-1,4-*N*-acetylglucosamine disaccharide; the expected product of cleavage of a NAG oligosaccharide substrate. The other structure has no ligand and was solved in space group C222_1_ with a single chain in the asymmetric unit (Apo) (PDB 4WLI). The apo structure forms a similar dimer to that seen in the R3 structure (in this case with the dimeric symmetry axis corresponding to the crystallographic two fold axis parallel to the unit cell b axis). Details of the data collection and refinement are given in Table 1.

**Table 1:** Data collection and refinement statistics

Crystal	+NAG (truncated K32-E218)	Apo (P73-E218)	+anhydro (R52-K217)
PDB CODE	4WJT	4WLI	4WLK
Data collection			
Wavelength (Å)	0.9795	0.9200	0.9795
Space group	R3:H (146) (Hexagonal Cell)	C222_1_ (20)	R3:H (146) (Hexagonal Cell)
Cell dimensions			
*a*, *b*, *c* (Å)	145.5, 145.5, 37.8	50.0, 117.3, 61.0	147.2, 147.2, 37.9
α, β, γ (°)	90, 90, 120	90, 90, 90	90, 90, 120
Resolution (Å)^a^	36.37–1.21 (1.23–1.21)	30.8–1.76 (1.79–1.76)	42.50–2.03 (2.08–2.03)
Total number of reflections ^a^	290513 (14071)	132867 (7892)	74023 (5113)
Number of unique reflections ^a^	89967 (4420)	18150 (1026)	19631 (1439)
R_merge_^a^	0.043 (0.569)	0.098 (0.745)	0.122 (0.576)
R_meas_^a^	0.060 (0.792)	0.114 (0.860)	0.162 (0.764)
R_pim_^a^	0.041 (0.549)	0.057 (0.425)	0.106 (0.497)
CC (1/2) ^a^	0.998 (0.607)	0.998 (0.771)	0.983 (0.620)
Solvent content (%)	45.7	53.3	44.0
Molecule/asymmetric unit	2	1	2
Wilson B-factor (Å^2^)	14.4	17.3	21.4
I/σI ^a^	10.3 (2.4)	13 (2.5)	7.5 (2.2)
Completeness (%)^a^	98.8 (97.2)	99.8 (99.6)	99.4 (98.7)
Redundancy ^a^	3.2 (3.2)	7.3 (7.7)	3.8 (3.6)
Refinement			
Resolution (Å)^a^	36.39–1.21 (1.23–1.21)	30.798–1.76 (1.85–1.76)	42.51–2.03 (2.14–2.03)
Reflection, working	85213	18131	19628
Reflection, free	4468	927	968
R_work_/R_free_ (%)	11.1/14.4	16.2/20.2	16.76/20.80
No of non-H atoms	2815	1264	2506
Protein	A: 1185 B: 1185	1120	A: 1139 B: 1133
Others	45 (NAG)	16 (EDO)	56 (1,6-anhydro-disaccharide)
	13 (Polypropylene glycol)		
	16 (DMSO)		
Water	371	128	178
B factors (Å^2^)^b^	24.0	26.6	32.4
Protein	A: 21.7 B: 21.6	25.5	A: 33.1 B: 31.2
Others	20.3 (NAG)	35.1 (EDO)	28.0 (1,6-anhydro-disaccharide)
	44.5 (Polypropylene glycol)		
	64.6 (DMSO)		
Water	37.0	35.05	36.839
Rmsds			
Bond lengths (Å)	0.023	0.016	0.005
Bond angles (°)	2.2	1.495	0.990
Ramachandran plot			
Favoured (%)	98.1	97.8	95.4
Allowed (%)	1.8	2.4	4.6
Outliers (%)	0	0	0

^a^ Values in parentheses are for the highest-resolution shell

^b^ Average over all atoms

$$ {R}_{merge}=\frac{{\displaystyle {\sum}_{hkl}}{\displaystyle {\sum}_j}\left|{I}_{hkl,j}-\left\langle {I}_{hkl}\right\rangle \right|}{{\displaystyle {\sum}_{hkl}}{\displaystyle {\sum}_j}{I}_{hkl,j}} $$

$$ {R}_{meas}=\frac{{\displaystyle {\sum}_{hkl}}\sqrt{\frac{n}{n-1}}{\displaystyle {\sum}_{j=1}^n}\left|{I}_{hkl,j}-\left\langle {I}_{hkl}\right\rangle \right|}{{\displaystyle {\sum}_{hkl}}{\displaystyle {\sum}_j}{I}_{hkl,j}} $$

$$ {R}_{\mathrm{p}.\mathrm{i}.\mathrm{m}}=\frac{{\displaystyle {\sum}_{hkl}}\sqrt{\frac{1}{\mathrm{n}-1}}{\displaystyle {\sum}_{j=1}^n}\left|{I}_{hkl,j}-\left\langle {I}_{hkl}\right\rangle \right|}{{\displaystyle {\sum}_{hkl}}{\displaystyle {\sum}_j}{I}_{hkl,j}} $$

where *I*_hkl_ is the reflection intensity and < *I*_hkl_ > is the average intensity for multiple measurements of that reflection.

### Fold analysis

**Fig. 1:**
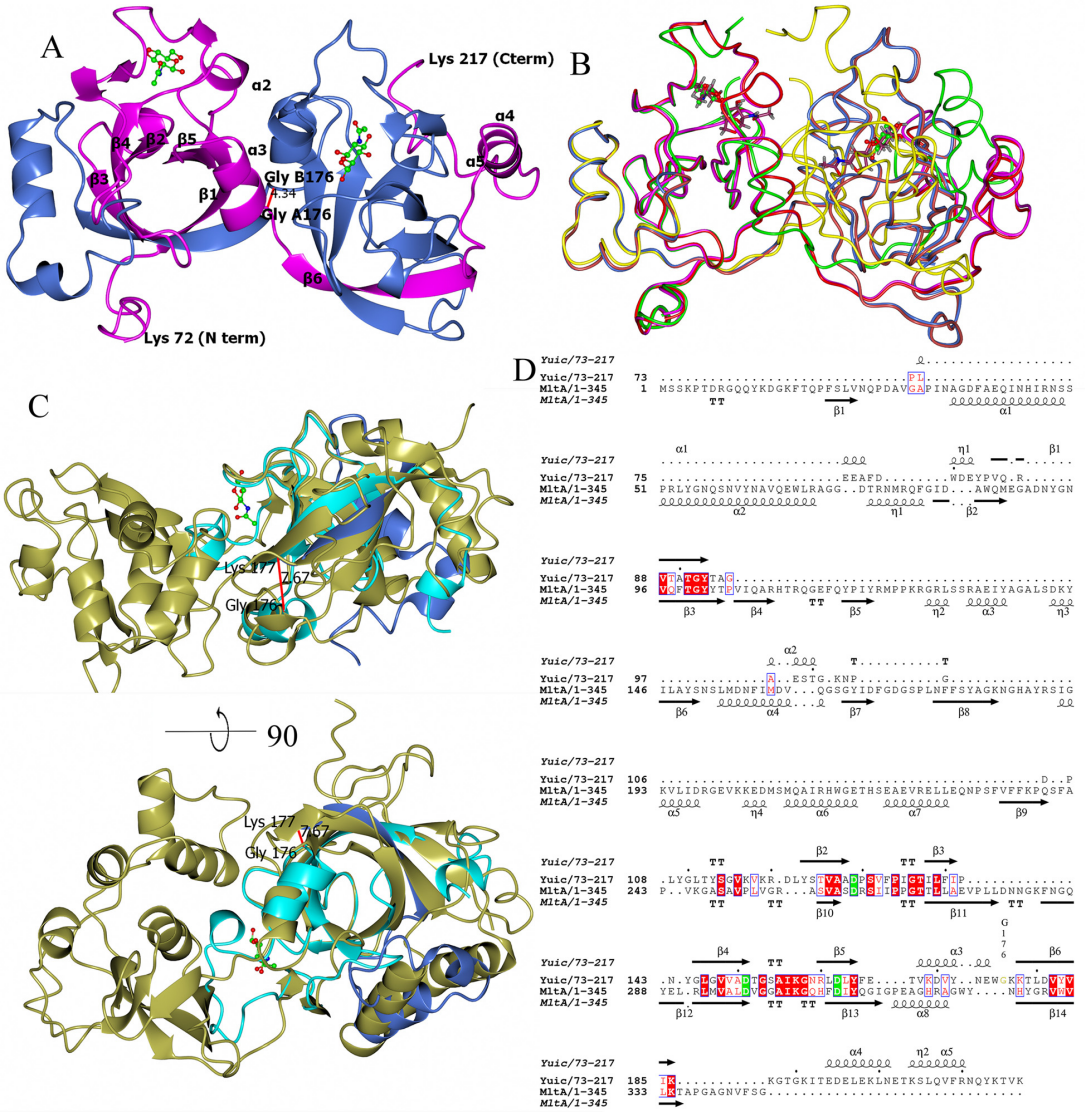
Structure of YuiC and comparison with MltA. **a** Dimer of YuiC with NAG bound. Chain A is in magenta and Chain B in blue with the positions of starts and ends of secondary structure elements labelled. NAG is ball and stick with carbon in green, oxygen in red and nitrogen in blue. The distance between the CA of G176 of each chain is shown in Å. **b** Structural Superposition of YuiC structure backbones. +NAG chains in magenta and blue with ligand in green, +Anhydro chains in red and pale crimson and ligand in dark purple. Apo chains in green and yellow. **c** Structural superposition of + NAG YuiC in cyan (A72-176) and blue (B177-217) (pseudo monomer) and MltA from *E.coli* (PDB 2ae0) [19] in gold. Lower picture is 90° rotation around horizontal of upper. The distance between A176 and B177 of YuiC is shown in Å. **d** Sequence alignment based on the structural superposition in C with secondary structure elements labelled, conserved aspartates shown in green and other conserved residues shown in red. G176, where the domain swap is centred, is coloured yellow and labelled. Structural superposition used SSM [36] in CCP4MG [37], structural alignment generated by UCSF chimera [38], structures drawn with CCP4MG [37] and alignment with ESPRIPT [39]

The overall fold of each domain of the symmetric dimer consists of a mixed direction six stranded double psi beta barrel surround by five helices (Fig. 1a). In all the structures, the final two helices and the last strand of the beta barrel of each half are supplied by the other chain in the dimer. This appears to be a classical example of crystallographic (sub)domain swapping as defined by Eisenberg [17]. It is likely, and certainly topologically possible, that in the monomeric form seen in solution the last strand (β6) and final helices (α4 and α5) of the domain are provided by the same chain. These three secondary structural elements then swap to form a dimer at higher protein concentrations (Fig. 1a).

The domain swapping is probably enabled by the ability of G176 to adopt the necessary phi-psi angles to allow either monomer or dimer to form. G176 lies at the end of the third helix. The CAs of G176 in the two chains are only 4.34 Å apart (Fig. 1a), so movement of a single helix to an “average” position in the monomer is plausible. Most of the dimer interface interactions would be found in a monomer, the exceptions are the interface between the two copies of the third helix (residues 166–178). Taking just the residues 166–178 from the two chains of + NAG using PISA [18] 316 Å^2^ is buried between the two helices and surrounding linkers with no additional hydrogen bonds or salt bridges definitely formed in the dimer compared to two separate monomers. This compares to 3955 Å^2^ in the total interface between the A and B chains. Two hydrogen bonds are formed from residues in this swapping region to other parts of the second chain. E166 side chain forms a hydrogen bond to T 215 very close to C-terminus of the other chain, and would presumably maintain this link in the monomer. The hydroxyl of Y172 forms a hydrogen bond to Glu98 in the other chain. It would take a movement of about 5 Å, rotating the helix in the right direction to bring G176 to the position of G176 in the other chain, for this to be an intrachain rather than an interchain hydrogen bond. This indicates that the dimer should only be slightly more stable than the monomer.

The two ligand bound structures have good electron density for all residues, and superimpose very well with a RMSD 0.40 Å over 289 CA atoms of the dimer or 0.23 Å over 145 CA atoms of the pseudo monomer (formed by chain A 72–176 and chain B 177–216) (Fig. 1b). The apo structure is locally less ordered than the substrate bound structures, lacking density for residues 97–100 and for four residues at the C-terminus compared to the + NAG structure, which just misses one residue at the C-terminus. Otherwise, the apo form of the pseudo monomer superposes well onto the + NAG structure with an RMSD of 1.15 Å over 135 CA atoms for the pseudo monomer. The domain swapped dimers superimpose less well with the RMSD for the apo dimer vs + NAG of 2.81 Å over 241 CA atoms as a consequence of flexibility in the positioning of the pseudo monomers within the dimer. The domain swapped dimer corresponds to a two-fold axis parallel to the third helix which lies at the centre of the dimer interface. In the apo crystals, the two fold axis is crystallographic.

If one pair of pseudo monomers is superimposed between apo and + NAG, it requires a rotation of 26° and a translation of 6 Å along a screw axis perpendicular to the third helix to superimpose the second pair of monomers (Fig. 1b) starts at residue 167 just before the third helix, however the two residues with very large phi/psi angle changes between liganded and apo structures, where most of the movement arises are G176 (phi/psi + NAG −83/−25 apo −77/169) and K178 (phi/psi + NAG −139/135 apo −88/−40). The flexibility of G176 agrees with, but does not prove, our proposal that a major rearrangement at this residue will generate the non-domain swapped monomer. The NZ of K178 in the apo structure forms hydrogen bonds to both the main chain and side chain carbonyls of N173, whereas in the liganded structures this side chain is pointing in to solvent. Whether this is the cause of the phi/psi angle at this residue is not clear. The region 176–178 lies away from the sugar binding site, so the change is not a direct result of ligand binding. However the other end of the third helix lies quite close to the ligand binding site and the loop that becomes ordered on ligand binding, so the difference in domain position may be propagated from sugar binding. However, it is also possible that changes in crystal packing may be the sole cause of the difference in the position of the second monomer seen in the apo structure.

### Comparison to MltA

MltA (membrane bound lytic transglycosylase A) (PDB 2ae0) [19] is defined as a single domain in SCOP, but as two domains in CATH - (2.40.40.10) the Barwin-like endoglucanase beta barrel, formed by a section from the N terminus (residues 20–104) (strands β1-3) and the C terminal region (243–337) (strands β10-14), and an unclassified domain (105–242). MltA superimposes on the YuiC (+NAG) structure with an RMSD of 2.07 Å over 102 of the 146 modelled residues (72–217) in the pseudo monomer (Fig. 1c). This is entirely within the beta barrel domain of MltA, which is 180 residues long and is slightly longer than the ordered region of YuiC (146 residues). Overall YuiC resembles closely the Barwin-like endoglucanase beta barrel of MltA, but instead of the second 138 residue domain of MltA, YuiC just has a 10 residue loop linking the two sections of the barrel. All the beta strands of YuiC have equivalents in MltA (Fig. 1d). The first strand and adjacent peptide of YuiC (84–96) is overlapped by the second and third strands of the MltA (82–104), which has an eight residue loop between the two strands that does not superimpose with YuiC. There is no equivalent of the first strand and helix of MltA in YuiC. Where the CATH Barwin-like endoglucanase domain of MltA begins again (243–253), the backbone is very close to YuiC (110–120) in a small hairpin in both structures. The second to fifth strands of YuiC superimpose with the tenth to thirteenth of YuiC. Before the final strand of the beta barrel both have helices, which do not superimpose well. The YuiC helix at this point, α3, is where the domain swap begins. The final strand of the beta barrel (β6) is formed by the last strand of YuiC from the other chain in the domain swap and is equivalent to the last strand (β14) of MltA. YuiC then has a pair of helices, which are close in space to the helices at the N-terminus of MltA but are not structurally equivalent.

### Ligand binding

**Fig. 2:**
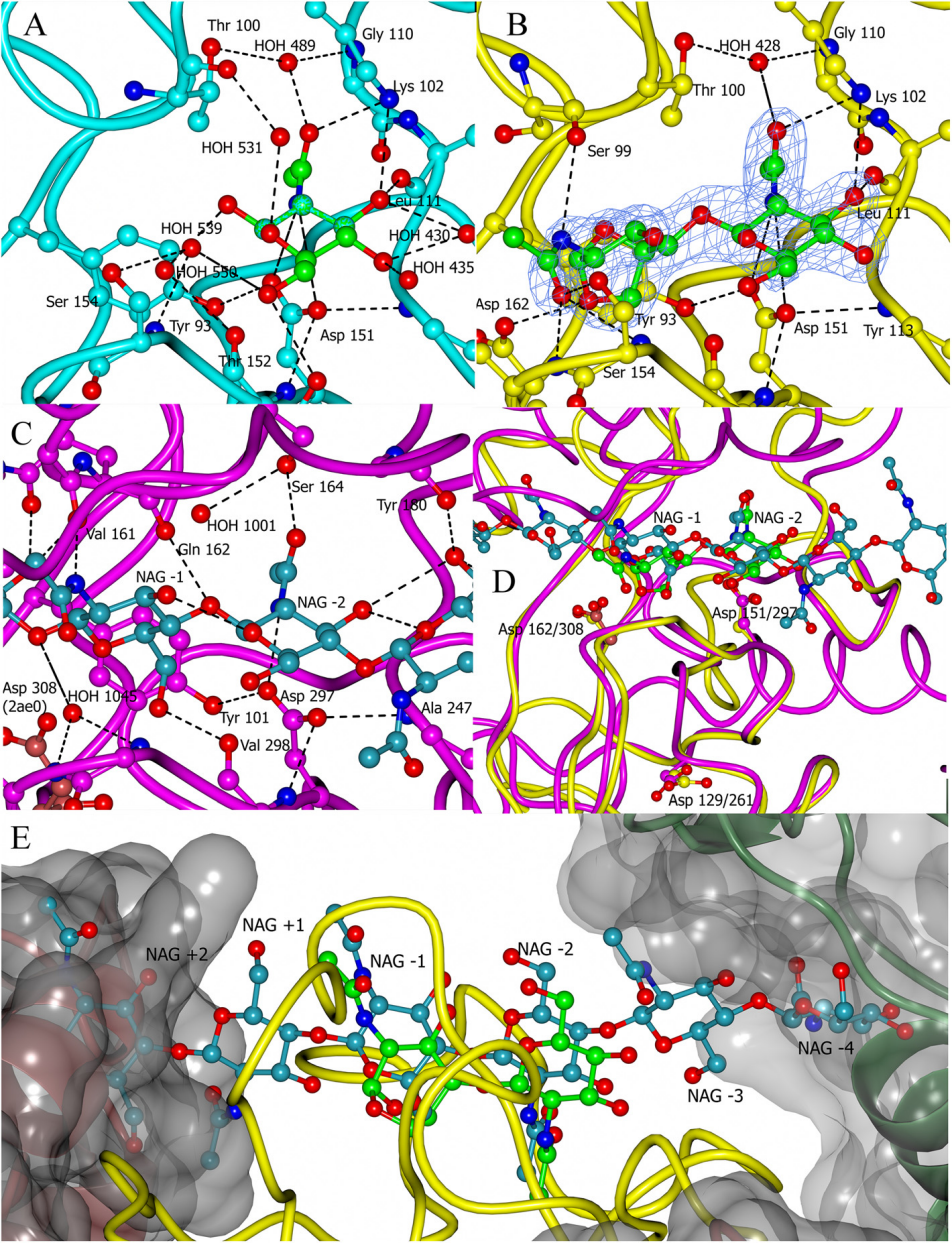
Interactions with ligands for YuiC and MltA. **a** Interaction of YuiC (*cyan*) with NAG (*green*) (H bonds shown as *black dotted lines* and non-carbon atoms *O red* and *N blue*). **b** Interaction of YuiC (*yellow*) with 1,6-anhydrodisaccharide (*green*). 2Fo-Fc electron density for the ligand at 1.0 sigma shown clipped to 1.5 Å around the ligand. **c** Interaction of MltA (*magenta*) with hexachitose (*dark cyan*) (PDB 2pi8) [20]. 2pi8 is a D308A mutation to prevent catalysis so D308 (*light crimson*) from the superposed active MltA (PDB 2ae0) is shown [19]. **a-c** are superimposed views **d** superposition of YuiC (*yellow*) with MltA (*magenta*) showing the superposition of substrates hexachitose (*dark cyan*) and the 1,6-anhydrodisaccharide (*green*). This shows the ligands overlapping at the −1 and −2 sites. The sidechains of the three conserved aspartates giving rise to the 3D domain name are also shown. **e** The potential clash of the ends of a hexachitose in the YuiC structure showing the +2 NAG of MltA (2pi8) clashing with chain B domain swapping helix (*dark crimson* helix and *transparent grey* surface) and the −4 NAG clashing with a symmetry related copy of YuiC in the lattice (*dark green* and *transparent grey* surface). Diagrams drawn with CCP4mg [37]

Crystallisation of YuiC in the presence of NAG gives a structure with a single well defined NAG per chain. Incubation of YuiC with 5 mM penta-NAG in the crystallisation results in two linked sugars in the final structure per chain. One of the sugars is a NAG that occupies the same −2 site as the sugar in the crystals grown in the presence of NAG monomer. The second sugar has clearly formed a 1,6-anhydro reaction product. The interactions of these compounds with YuiC are shown in Fig. 2a and b and discussed more fully below. Peptidoglycan consists of chains of alternating N-acetylglucosmine (NAG) and N-acetylmuramic acid (NAM) sugars, cross-linked with peptide chains. The two sugars differ at the O3 position, where NAM has a lactate, which then links to the crosslinking peptide, whereas NAG just has an OH. This means that NAM is much more bulky at the O3 position, which often confers the selectivity in cleavage.

The structure of the catalytically inactive D308A MltA with chitohexose [20] (PDB 2pi8) has six clearly defined NAG sugars, four, −4 to −1, on the non-reducing end before the cleaved bond and two, +1 and +2, at the reducing end. The NAG in + NAG superimposes with the −2 position in the chitohexose in MltA. The anhydro sugar lies at the −1 position and the unmodified NAG lies at the −2 position in the disaccharide reaction product. The interaction of the conserved D297 (MltA)/D151 (YuiC) with the N of the *N*-acetyl group of the NAG at the −2 position is conserved (Fig. 2a-c, Table 2). Further hydrogen bonds to this sugar in YuiC are from the non-conserved K102 NZ to the N-acetyl carbonyl and O3 of NAG. MltA does not have any atoms near K102 NZ in the superposition and K102 lies in the YuiC insert that replaces a whole domain in MltA. The OH of S164 in MltA does form an H bond to the −2 N-acetyl carbonyl, but lies 4.2 Å from K102NZ in the superposition and does not also interact with O3, and there is a water (HOH 428) in the YuiC structure at the position of the MltA S164 OH.

**Table 2:** List of Hydrogen bonds between protein and substrates and conservation in MltA

Sugar Site	Ligand Atom	+NAG (4wjt)	+Anhydro (4wlk)	MltA (2pi8)	Conserved YuiC vs MltA
−2	O1	Thr152 O *via* HOH A550	None	(O4 of −1) Gln162 O	No. Thr100 YuiC is closest to MltA Gln162
−2	O3	Leu111 O	Leu111 O	None	No
−2	O3	Lys102 NZ	Lys102 NZ		No
−2	O4	HOH A430/435	None	None	
−2	O5	Thr100 O *via* HOH A531	HOH416 is too far (3.7 Å)	None	
−2	O6	Ser154 N and OG *via* HOH 539	None	None	
−2	O7	Lys102 NZ	Lys102 NZ		
−2	O7	Gly110 N and Ala 95 O *via* HOH A489	Gly110 N and Ala 95 O *via* HOH A428	Ser164 OG	No. HOH 428 and Ser164 OG are close
−2	N2	Asp151 OD1 and OD2	Asp151 OD1 and OD2	Asp297 OD2	Yes. Asp151/297
−1	O1		Asp162 OD2	(O4 of +1) none but Asp308 mutated to Ala	Yes. Asp162/308
−1	O3		None	None	
−1	O5		None	None	
−1	O6			Val298 O	
−1	O7		Ser 154 N and OG Ala 155 N	Gly300 N and Ala 301 N *via* HOH A1045	Yes, but contact longer *via* water in MltA
−1	N2		Ser99 O	Val161 O	No. Close in space but not conserved

More generally there is good conservation of the backbone on the D151 side of the sugar, but little conservation on the other face. The O3 hydroxyl in the −2 position forms a hydrogen bond to the backbone carboxyl of L111, which would prevent there being a N-acetylmuramicacid (NAM) at this position. NAM can easily be accommodated at the −1 position as the O3 of the sugar, which has the lactic acid group in NAM and then the peptide in peptidoglycan, is pointing into the solvent. The −1 site in MltA has a hydrogen bond to the main chain carboxyl of residue V298 from the O6 hydroxyl (Fig. 2c, Table 2). This backbone position is conserved in YuiC, but the hydroxyl has moved away to form the anhydro product and so this contact is lost in the product. The N of the *N*-acetyl group is interacting with the main chain carboxyl of V161 in MltA. This is roughly equivalent in position to the carbonyl of S99 in YuiC + anhydro structure, which forms a similar interaction, despite the overall fold not being conserved in this region. The carbonyl oxygen of the acetyl group of the −1 sugar in the YuiC + anhydro product interacts with two main chain NH groups (S154 and A155), which are conserved in MltA (G300 and A301), although the carbonyl also interacts with the side chain OH of S154 in YuiC, which is an extra interaction compared to MltA. In MltA the acetyl carbonyl is further away and the interaction with G300/A301 is water mediated. Without a product structure for MltA or an uncleaved substrate in YuiC it is impossible to determine whether the differences in binding are due to substrate/product differences or protein differences.

The superposition of the MltA sugars allows us to look more widely at possible sugar binding sites in YuiC. Intriguingly there is only room for one sugar site on the vacant + side of the cleavage in the + NAG and anhydro structure. The +2 sugar in the MltA superposition clashes with the third (domain swap) helix backbone (Fig. 2e). This would prevent the sugar chain being longer than +1 and so the dimer could only remove a terminal NAG (ie be an exo glycosidase). However the movement of the second pseudo monomer of the domain swapped dimer in the apo structure described above, displaces the third helix away from this position so that this clash is reduced to ends of side chains, which could adopt other positions. This probably would allow cleavage within a chain (endo) in the dimer as well, and certainly allow removal of disaccharides as seen in many lytic transglycosidases. In the pseudo monomer, the block from the helix probably does not occur and the active site is much more open so the monomer is likely to be able to cleave in either an endo or exo mode.

The main interactions with the +1 sugar in MltA are formed by residues V161 and Q162, which lies in the inserted domain in CATH that is not present in YuiC. However, the hydroxyl of S99 of YuiC, which is part of the much more direct link that replaces the inserted domain, lies close to the position of the Q162 side chain of MltA and could potentially hydrogen bond to O3 of the +1 sugar. There is not much space round the superimposed +1 O3 hydroxyl in YuiC and so the +1 position is likely to be specific to NAG and not able to house a NAM residue.

Although we can only clearly see two sugars in the product, potentially a third may be present in the anhydro product in a disordered state. The regions of MltA that interact with the −3 and −4 sugars in MltA are not homologous with YuiC. The superimposed −4 sugar of MltA collides with a symmetry related molecule of YuiC (Fig. 2e) suggesting that a product with four sugars would not bind in the lattice. Intriguingly this would be the obvious product of the penta-NAG in the YuiC dimer R3 crystal as the +2 position is also sterically blocked. It is hard to envisage YuiC having any positive interaction with a sugar in the −4 position as the protein does not extend out that far. Despite being larger, MltA only has limited interaction with the sugar at the −4 position. Careful inspection of the −3 site indicate some waters are in positions likely to be where hydroxyls of the sugar would be positioned, but if it is present the sugar is either much more mobile or much less occupied due to cleavage at a mixture of positions in the penta-NAG. It is more likely that multiple cleavage events before crystals formed have led to a predominant two sugar anhydro product and this form bound to the dimer may have been preferentially selected by the lattice.

### Catalytic activity

The conserved aspartates of the 3D domain, the Pfam annotation of YuiC (http://pfam.xfam.org/family/3D), superimpose well with the equivalent residues in MltA. YuiC D162 is equivalent to MltA D308 (A308 in 2pi8); YuiC D129 to MltA D261and YuiC D151 to MltA D297. No equivalent atoms are further than 1.4 Å apart and all CAs within 0.5 Å.

**Fig. 3:**
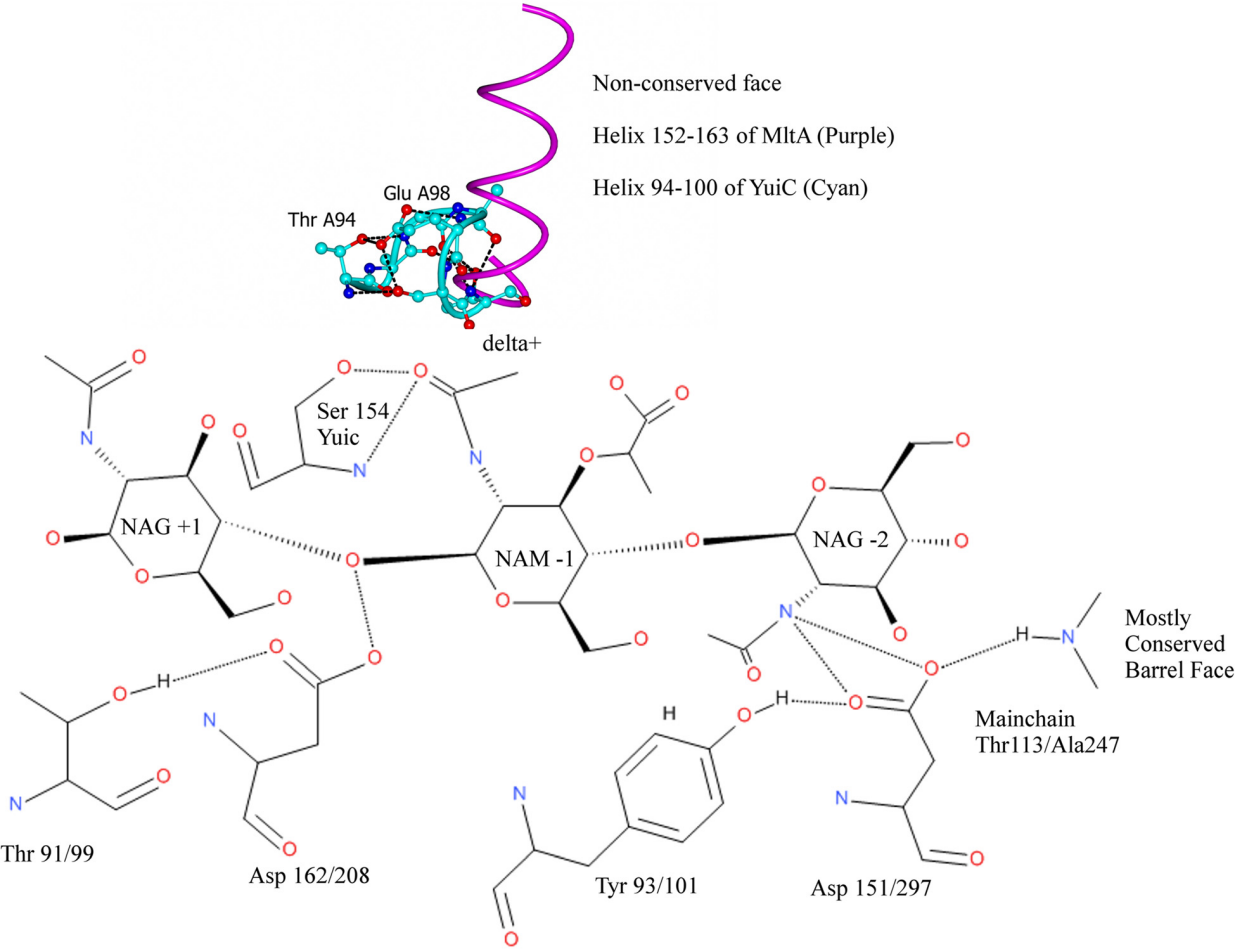
Schematic of the YuiC active site showing the conservation with MltA. The lower barrel side shows significant conservation to MltA including the conserved catalytic aspartate and the residues allowing mechanism 2 of Powell *et al.* [21]. The upper face is not conserved. Substrate assisted catalysis is unlikely because S154, which is unique to YuiC is holding the acetyl carbonyl in the wrong place for this mechanism. The helix from MltA (*purple*) is thought to stabilise the carbenium ion. It is possible that the helix from YuiC (*cyan*) may play a similar role or release E98 to act as a second carboxylate, as 97–100 are disordered in the apo structure indicating flexibility in this region. The two helices are shown in their superimposed positions. Where two numbers are given the first is *B.subtilis* YuiC and the second *E.coli* MltA (2pi8 numbering)

The conserved D162 is the catalytic carboxylate and is orientated by T91 which is conserved in MltA (T99) (Fig. 3). A number of proposed mechanisms for MltA have been put forward. In the preferred mechanism of Van Straaten *et al.* [20] the catalytic aspartate residue is proposed to protonate the leaving hydroxyl at the +1 position and deprotonate the O6 hydroxyl, which attacks a carbenium ion intermediate to form the anhydro product. It is proposed in MltA that the carbenium ion is stabilised by the α4 helix dipole. This helix lies in the inserted domain which has no equivalent in YuiC. However in the + NAG and anhydro product structures the nearest residues to the position of the helix in the superposed MltA are E98 and S99. The main chain carboxyl of S99 is hydrogen bonding to the N of the *N*-acetyl group of the −1 anhydrosugar with the side chain of E98 pointing away towards the side chain of T94. However the stretch of residues from 97 to 100 is disordered in the apo structure indicating that these residues are flexible and therefore E98 may be able to rearrange and act as a second carboxylate in the reaction mechanism, either just to stabilise the carbenium ion as the dipole is proposed to do in MltA, or opening up the possibility of a two carboxylate mechanism analogous to the retaining lysozymes.

Substrate assisted catalysis has also been proposed for MltA [21]. This would require the −1 sugar N-acetyl oxygen to be on the opposite face of the substrate from the O6 that generates the anhydrosugar. However in YuiC the −1 sugar N-acetyl group oxygen of the anhydro product is interacting with S154 and is on the same face as the O6 oxygen would be. Unless the S154 interaction is only formed in the product then substrate assisted catalysis using the N-acetyl group is unlikely, as a very large rearrangement of the N-acetyl group is required to place it in position to assist in substrate catalysis from its position in the product structure. All the homologous residues for the second mechanism of Powell *et al.* [21] involving Y93 and D151 of YuiC acting in the same way as proposed for Y140 and D393 of *N. gonnorrhoeae* MltA to abstract the proton from the O6 to promote nucleophilic attack on the carbenium to form the anhydro product.

### Is the monomer or the dimer the true structure?

**Fig. 4:**
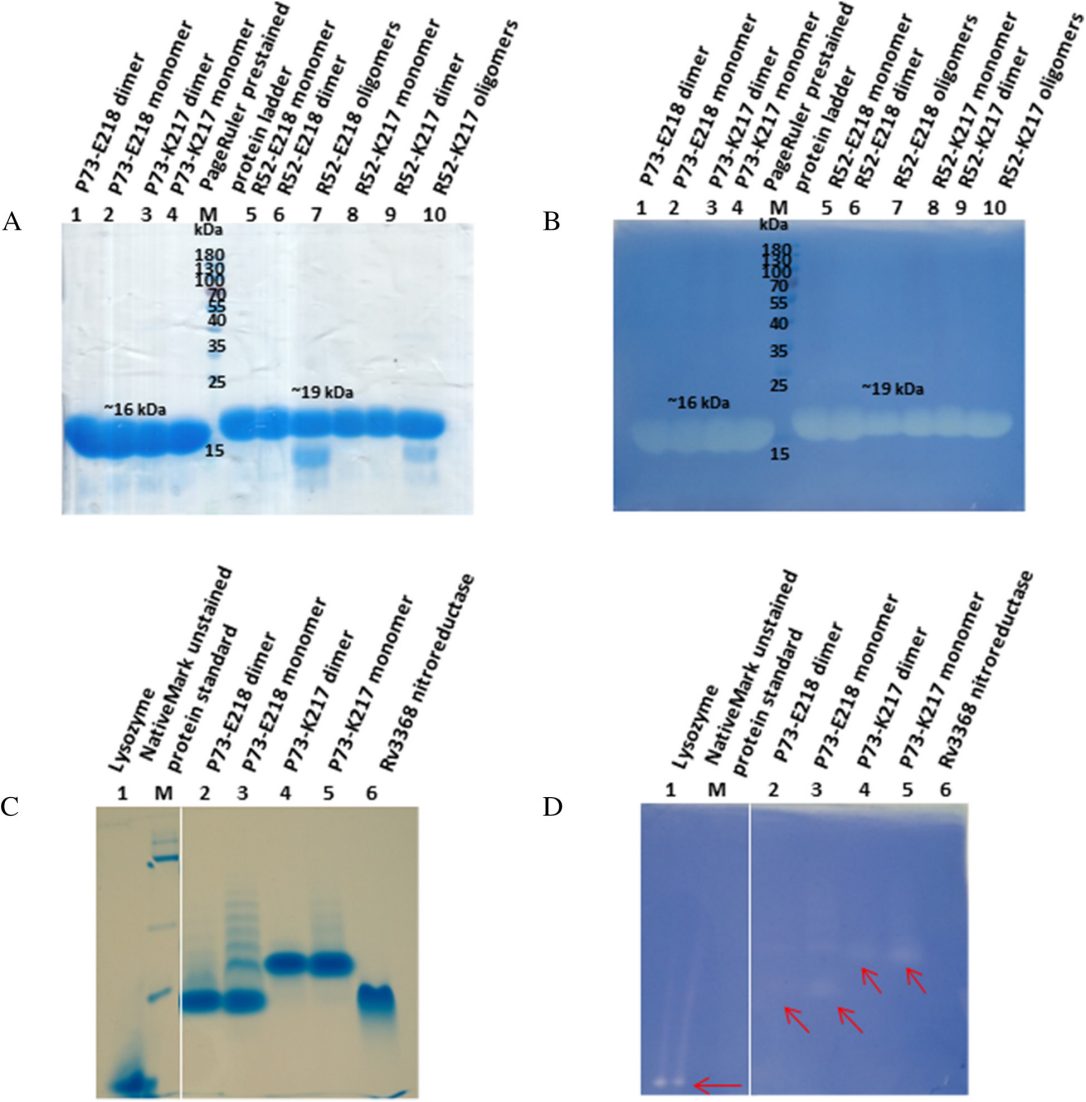
Zymograms of YuiC. **a** SDS-PAGE and **b** denaturing zymogram of YuiC_P73 and YuiC_R52 constructs. Lane 1: YuiC_P73-E218 dimer, 2: YuiC_P73-E218 monomer, 3: YuiC_P73-K217 dimer, 4: YuiC_P73-K217 monomer, M: PageRuler prestained protein ladder, 5: YuiC_R52-E218 peak 1 (monomer), 6: YuiC_R52-E218 peak 2 (dimer), 7: YuiC_R52-E218 peak 3 (oligomers), 8: YuiC_R52-K217 peak 1 (monomer), 9: YuiC_R52-K217 peak 2 (dimer), 10: YuiC_R52-K217 (oligomers). **c** Native-PAGE and **d** native zymogram of YuiC_P73 constructs. Lane 1: Lysozyme, M: NativeMark unstained protein standard, 2: YuiC_P73-E218 dimer, 3: YuiC_P73-E218 monomer, 4: YuiC_P73-K217 dimer, 5: YuiC_P73-K217 monomer, 6: negative control (Rv3368). Positions of the principal bands in the native zymogram are marked with *red arrows* as they are faint

Zymograms indicate that protein from both peaks of the gel filtration are enzymatically active and can degrade peptidoglycan after refolding after SDS-PAGE (Fig. 4a-b). However, this does not demonstrate which oligomeric states are active as the refolding from the unfolded monomer in the gel could have led to either, or a mixture of both, oligomeric states. A native gel shows no major difference in apparent size or activity of the samples originating from the monomer and dimer peaks (Fig. 4c-d), probably the very high concentration in the stacking gel has driven the protein largely into the dimer state. There are some other higher oligomer bands that are also active, particularly from the monomer peak.

The domain swapped dimer seen in the crystal may be an artefact of high level expression in the *E.coli* cytosol and high concentrations used for structural studies, however, we have no direct evidence for this. Nevertheless, both the monomer and dimer are stable species. Rerunning samples of either peak, after being frozen for some weeks, gives a single peak with a similar retention volume on gel filtration as when first run (Additional file 1: Figure S1). This indicates that both states are stable and there is at best very slow kinetic interchange between the two.

It is pure speculation as to what the oligomeric state is in *B.sutbtilis in vivo.* A mixture of the two states is possible, particularly as both are kinetically stable and probably active. Highly expressing protein in the cytoplasm of *E.coli* is different from an unknown level of expression of secreted protein in *B.subtilis*, so the distribution seen in our experiments may not reflect nature. Furthermore YuiC may interact with the cell wall or other enzymes through the disordered region at the N-terminus, which could influence its ability to oligomerise. Peptidoglycan remodelling enzymes are known to interact. RpfB and RpfE bind to RipA and there is synergy in cleavage seen between the two [22–24]. Indeed RpfB and RipA assemble into a larger complex with PBP1 at the poles septum [24]. The assembly of multiple peptidoglycan enzymes is a theme also seen in Gram-negative *E. coli* [25].

## Conclusions

The structure of YuiC from *B. subtilis* has shown that the stationary phase survival (Sps) proteins are smaller versions of the MltA family of lytic transglycosylases. This structural simplification is analogous to that of the resuscitation promoting factors in Actinobacteria being reduced versions of lysozyme and Slt proteins [7]. Indeed there is conservation of ancillary domains between groupings of the Rpfs and the Sps and in some cases synteny in the surrounding operons [14].

Our structural work has shown that the Sps protein family is indeed homologous to MltA, but a more compact, perhaps minimal, version of the enzyme. We have also trapped an 1,6-anhydrosugar product in the active site, showing that formation of a 1,6-anhydrosaccharide is the product of the reaction. The analogy with the resuscitation promoting factors of Actinomycetes supports the role of these proteins in stationary phase survival of the Firmicutes.

## Methods

### Protein cloning, expression and purification

Full-length mature protein K32-E218 (Uniprot YUIC_BACSU residues 32–218) was cloned into vector pET151/TOPO (Novagen, Merck Millipore) with a Tobacco Etch Virus (TEV) Protease cleavable His-Tag. This vector results in GIDPFT on the N-terminus after the TEV protease cleavage. Truncated (R52-K217, R52-E218, P73-E218, P73-K217) YuiC proteins were cloned into pNic28-Bsa4 plasmid (supplied by Dr Opher Gileadi of the Structural Genomics Consortium), which is a modified pET28a plasmid that allows ligation independent cloning [26] and only adds a single serine to the N terminus after TEV cleavage.

Proteins were expressed in *E. coli* Rosetta 2 (DE3) (Novagen, Merck Millipore) using Terrific Broth by induction with 0.25 mM IPTG at 30 °C for 6 h after the culture had reached A600 of 0.6. The cells were harvested and resuspended in lysis buffer containing 0.1 M Tris–HCl pH 7.0, 0.5 M NaCl, 50 mM Imidazole, 2 mM βME, 2 mg/mL lysozyme and a cOmplete EDTA-free protease inhibitor cocktail tablet (Roche Applied Science, Switzerland). The cell suspension was sonicated on ice at 20 Watts for 4 min twice using 5 s interval pulses and the sample was treated with DNaseI for 30 min. The sample was then centrifuged at 48,000 × g for 60 min at 4 °C to obtain the supernatant. The protein supernatant was loaded onto a 5 mL HisTrap FF (GE healthcare, USA) column using a peristaltic pump, washed with 10 column volumes of binding buffer (0.1 M Tris–HCl pH 7.0, 0.5 M NaCl, 50 mM Imidazole, 2 mM βME) and the his-tagged YuiC protein was collected in the elution buffer (0.1 M Tris–HCl pH 7.0, 0.5 M NaCl, 500 mM Imidazole, 2 mM βME). The protein sample was then incubated with TEV protease (1 mg/mL) and dialysed against 4 L buffer containing 20 mM Tris–HCl, pH 7.5 and 2 mM βME at 4 °C overnight. The protein was passed through a HisTrapFF column to collect untagged YuiC protein.

The proteins were further purified by ion exchange and gel filtration, the conditions used varied for each construct. For full-length protein, the sample was dialysed again using 20 mM Tris–HCl pH 8.4 prior to anion exchange chromatography. A Resource Q column (1 mL) was pre-equilibrated with buffer containing 20 mM Tris–HCl, pH 8.4, 2 mM βME before protein loading and gradient of 20 Column Volumes to 100 % of buffer containing 20 mM Tris–HCl pH 8.4, 1 M NaCl, 2 mM βME was used for protein elution. To further purify the protein, size exclusion chromatography was carried out using a HiLoad Superdex 200 column pre-equilibrated with 20 mM Tris–HCl pH 8.0, 50 mM NaCl, 2 mM βME buffer. For YuiC P73-E218 and YuiC P73-K217, the anion exchange chromatography used 20 mM Bis-tris pH 7.0, 2 mM βME for the binding buffer and 20 mM Bis-tris pH 7.0, 2 mM βME, 1 M NaCl for the elution buffer. For size exclusion chromatography, 20 mM Bis-tris pH7.0, 150 mM NaCl and 2 mM βME buffer was used. For YuiC R52-E218 and YuiC R52-K217, cation exchange chromatography was carried out using an SP HP (GE Healthcare) column. For YuiC R52-E218 protein, the binding buffer used was 50 mM MES pH 6.5, 2 mM βME and for the elution buffer, 50 mM MES pH 6.5, 1 M NaCl, 2 mM βME elution buffer. Whereas for YuiC R52-K217 protein, 50 mM HEPES pH 7.5, 2 mM βME was used as the binding buffer and 50 mM HEPES pH 7.5, 1 M NaCl, 2 mM βME as the elution buffer. Size exclusion chromatography for YuiC R52-E218 protein was carried out in 50 mM MES pH 6.5, 100 mM NaCl, 2 mM βME buffer.

### Zymograms

The 15 % SDS-PAGE zymogram gel was prepared by the addition of 0.2 % (w/v) lyophilised *M. luteus* cells to a standard 15 % SDS-PAGE resolving gel. Protein samples (1.57 mM) were mixed with 4× SDS sample buffer without βME and were not heated before loading. The gel was run at 180 V for an hour. After electrophoresis, to remove SDS the gel was rinsed three times by shaking gently in 100 mL of distilled water for 20 min. The gel was then rinsed with 100 ml of renaturation buffer (50 mM sodium phosphate, pH 5.0) for 30 min and then changed to fresh renaturation buffer (100 mL) for overnight digestion at room temperature. Triton X-100 (1 %) was added to all of the renaturation buffers to keep protein from aggregation. The zymogram was stained with 0.1 % methylene blue in 0.01 % potassium hydroxide and destained with distilled water until a clear band could be seen against the blue background. For the native PAGE zymogram, both the gel and protein samples (1.57 mM) were prepared without any reducing or denaturing agents (SDS, βME) and were not heated prior to gel loading. 0.2 % (w/v) *M. luteus* lyophilised cells was added to the 10 % separating gel. After electrophoresis, the gel was incubated with buffer containing 20 mM Tris–HCl pH 7.0, 150 mM NaCl and 2 mM βME for overnight digestion. The gel was stained and destained as described for the denaturing zymogram. Lysozyme was used as positive control, whereas a nitroreductase from *Mtb*, Rv3368, was used as the negative control.

### Crystallisation

YuiC at concentrations of 12–16 mg/mL was used to set up crystallisation trials by sitting drop vapour diffusion set up with a Mosquito robot. The monomer and dimer peaks from gel filtration were always concentrated and used separately. Crystals were only obtained from dimer peak samples. The MIDAS screen [27] proved particularly useful in identifying conditions. The crystallisation and cryoprotection conditions of the reported structures are Apo: YuiC (P73-E218) (0.2 M ammonium chloride, 25 % (v/v) glycerol ethoxylate, 0.1 M HEPES pH 7.5; cryoprotectant: 20 % ethylene glycol), +NAG: YuiC (degraded K32-E218) with 5 mM NAG (48 % (v/v) polypropylene glycol P400, 0.1 M HEPES pH 6.0, 3 % (v/v) DMSO; no cryoprotectant required) and + Anhydro: YuiC (R52-K217) with 5 mM penta-NAG (penta-N-acetyl-chitopentaose, Seikagaku Corporation Japan) (40 % glycerol ethoxylate; cryoprotectant: 20 % ethylene glycol).

### Structure solution and refinement

A dataset of a crystal of YuiC grown in the presence of NAG was collected at Diamond Synchrotron beam line I02 in space group R3, with two chains predicted in the asymmetric unit. The structure was not readily solved by molecular replacement. A large number of models were generated based on the remote homology to MltA using a variety of structure prediction websites and hand truncation and a range of molecular replacement packages. Eventually a solution was found using ACORN [28], a programme designed for *ab initio* phasing, using a starting model based on PDB 2ae0, the structure of *E. coli* MltA [19]. The model was generated by the Phenix MR_Rosetta protocol [29] using an HHPred [30] derived alignment. However, this model did not give a convincingly buildable solution on the default settings in phenix MR_Rosetta. A rebuild of the ACORN map with Arp/wARP [31] gave a virtually complete model, which was further improved by cycles of rebuilding in coot [32] and refinement with Refmac5 [33]. The subsequent apo and + anhydro structures were solved by molecular replacement from the + NAG structure with phaser [34] and refinement with Refmac5 and phenix.refine [35] respectively. Residues 72–217 in both chains are modelled in + NAG, 72–216 in chain A and 73–216 in chain B of + anhhydro and 73–214, missing 97–100 inclusive, in the single chain of the apo structure.

### Availability of supporting data

The structures and structure factors are deposited in the RCSB Protein Data Bank as entries 4WJT (+NAG) http://www.rcsb.org/pdb/search/structidSearch.do?structureId=4wjt, 4WLI (Apo) http://www.rcsb.org/pdb/search/structidSearch.do?structureId=4wli and 4WLK (+anhydro) http://www.rcsb.org/pdb/search/structidSearch.do?structureId=4wlk. Additional figures are in a file supplementaryquayyuic2.pdf in PDF (Portable Document Format). It contains three figures and legends describing characterisation of oligomeric state by gel filtration, degradation of the full-length protein and NMR spectra showing loss of unfolded regions on degradation.

## Additional file

Additional file 1: Figure S1. Size exclusion chromatography molecular weight estimation of YuiC constructs. **Figure S2.** SDS-PAGE profile shows partial protein truncation of YuiC K32-E218. **Figure S3.** 1D NMR profiles of YuiC K32-E218.

## Abbreviations

βME: β-mercaptoethanol
EDTA: Ethylenediaminetetraacetic acid
IPTG: Isopropyl-β-D-thiogalactoside
MltA: Membrane-bound Lytic Transglycosylase A
*Mtb*: *Mycobacterium tuberculosis*
NAG: N-acetylglucosmine
NAM: N-acetylmuramic acid
NMR: Nuclear Magnetic Resonance
penta-NAG: penta-*N*-acetyl-penta-glucosamine
RMSD: Root Mean Square Deviation
Rpf: Resuscitation Promoting Factor
SDS: Sodium Dodecyl Sulphate
Slt: Soluble Lytic Transglycosylase
Sps: Stationary Phase Survival Protein
TEV: Tobacco Etch Virus
3D: Three Aspartate Domain
