# Supplementary material for: Structure of the stationary phase survival protein YuiC from B.subtilis
Source: BMC Struct Biol. 2015 Jul 11;15:12. doi: 10.1186/s12900-015-0039-z (PMC4499186; doi:10.1186/s12900-015-0039-z)
Supplement: Additional file 1: Figure S1. — Size exclusion chromatography molecular weight estimation of YuiC constructs. Figure S2. SDS-PAGE profile shows partial protein truncation of YuiC K32-E218. Figure S3. 1D NMR profiles of YuiC K32-E218. [file 12900_2015_39_MOESM1_ESM.pdf]

Supplementary Figure 1

A)

YuiC	Elution volume (mL)	Molecular weight (kDa)
K32-E218 dimer	84.44	48.11
K32-E218 monomer	92.66	24.31
Third peak	107.26	7.23
P73-E218 dimer	86.96	39.03
P73-E218 monomer	94.8	20.36
Rerun monomer	94.33	21.17
P73-K217 dimer	86.98	38.96
P73-K217 monomer	94.97	20.07
Rerun dimer	86.9	39.22

B) **Truncated YuiC K32-E218**

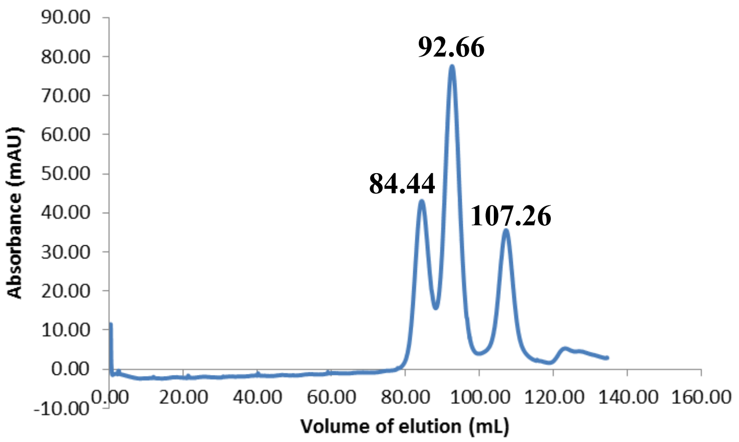

C) **YuiC P73-E218**

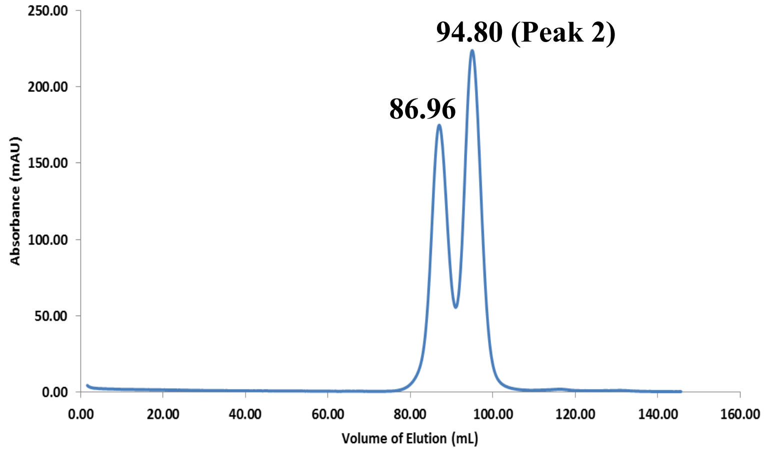

D) **Re-run monomer P73-E218 (Peak 2)**

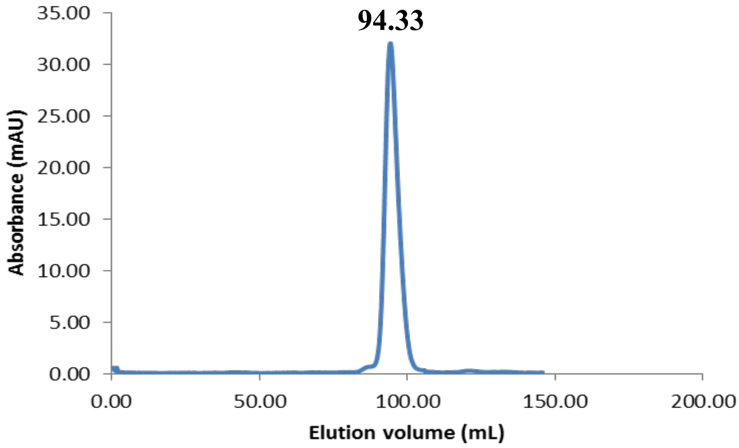

E) **YuiC P73-K217**

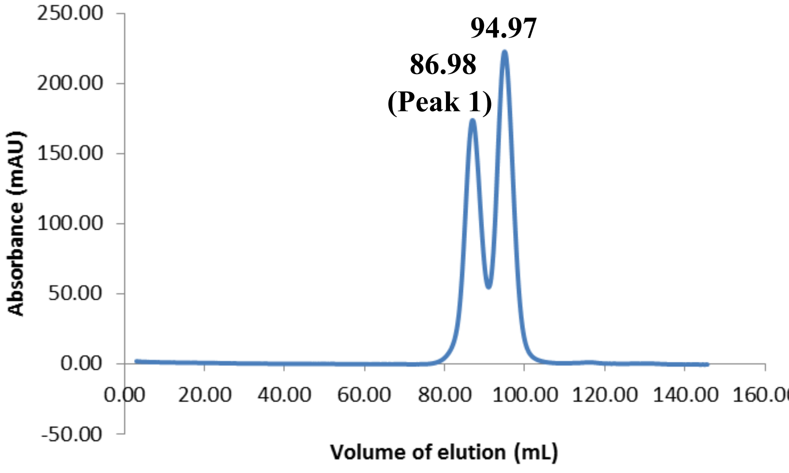

F) **Re-run dimer P73-K217 (Peak 1)**

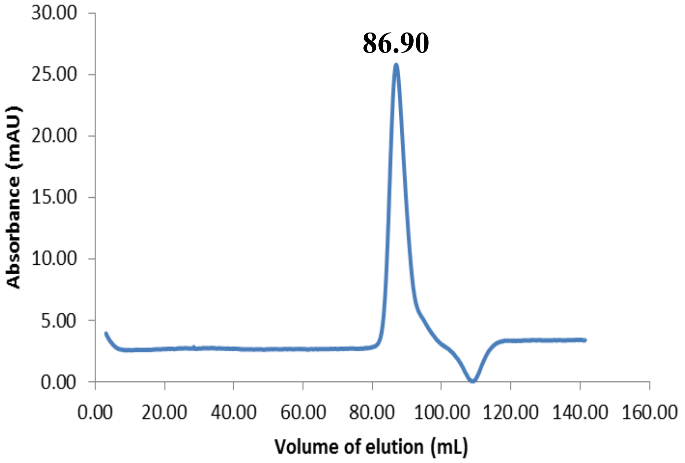

G)

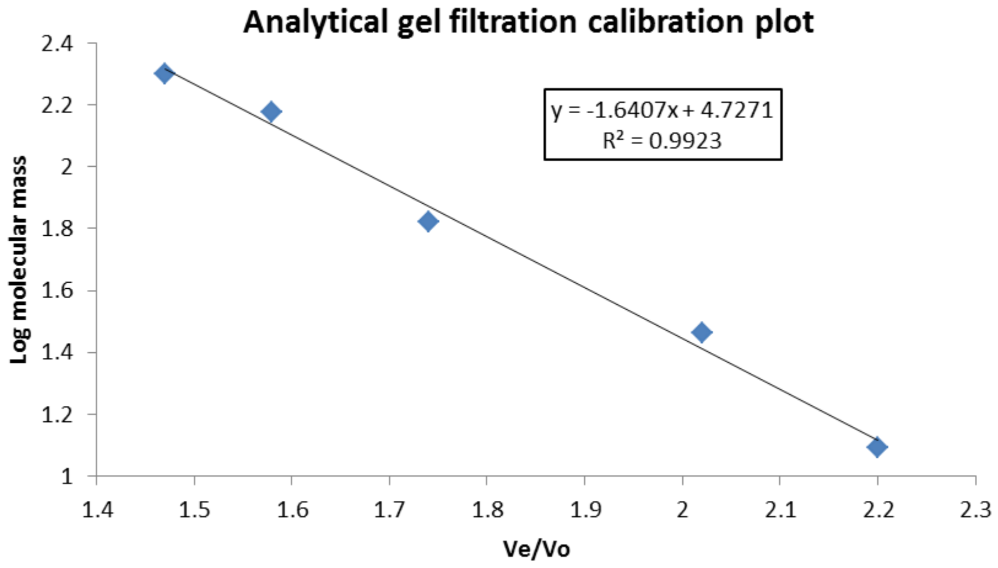

H)

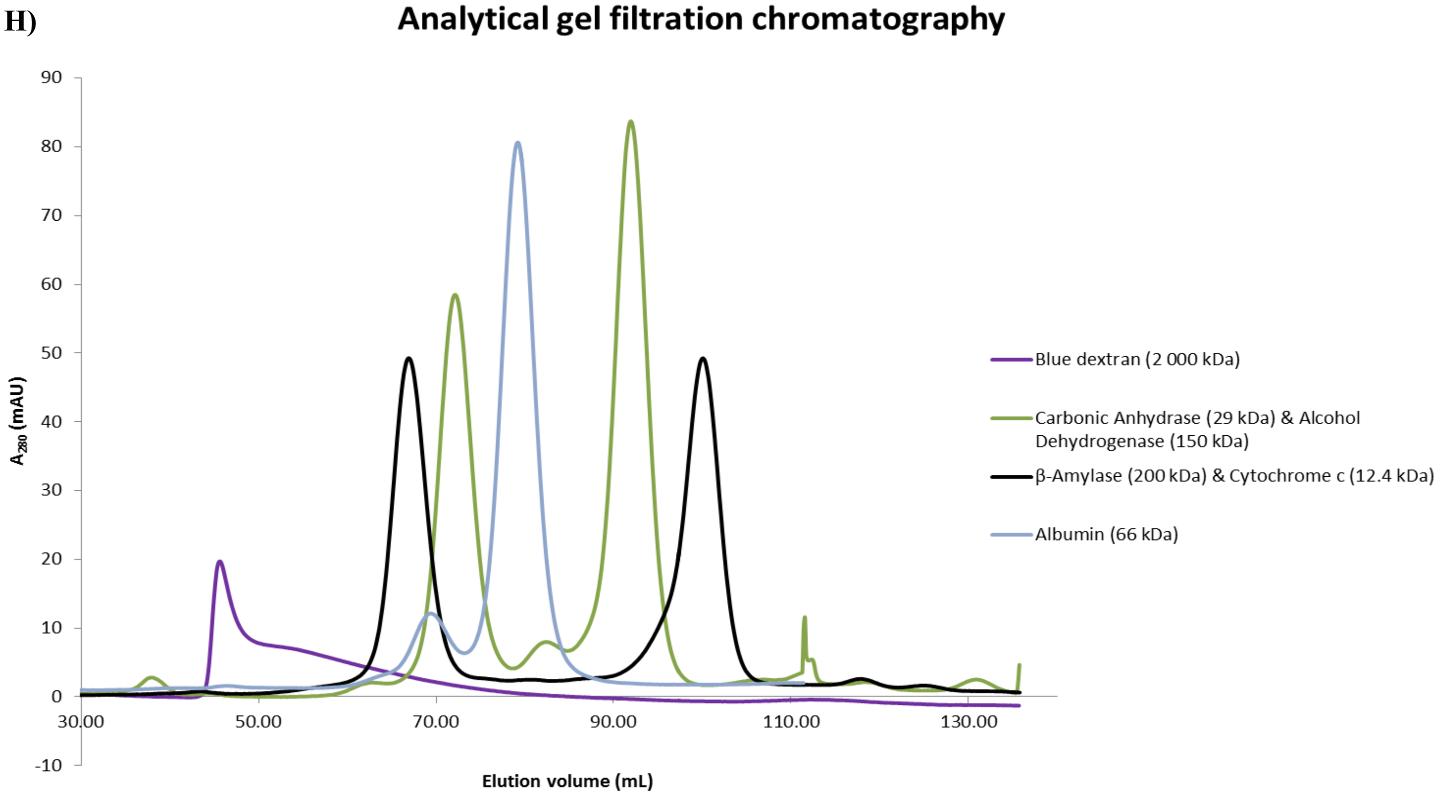

Figure S1. Size exclusion chromatography molecular weight estimation of YuiC constructs. A) Molecular weight estimation of each peak of YuiC calculated based on the elution volume against the analytical gel filtration calibration plot. B) Size exclusion chromatography profile of the truncated YuiC K32-E218. The three peaks correspond to a dimer (84.44 mL), monomer (92.66 mL) and the degraded product (107.26 mL). C) and E) Size exclusion chromatography profiles of YuiC P73-E218 and P73-K217 respectively, showing the dimer and monomer peak. D) and F) Re-run size exclusion chromatography of the monomer of YuiC P73-E218 and dimer of YuiC P73-K217, which still run as a monomer and dimer respectively. G) Analytical gel filtration calibration plot with its linear equation and R-squared value. H) Graph showing the column calibration of the Hiload 16/60 Superdex 200 column calibration using standards of known molecular weight.

Supplementary Figure 2

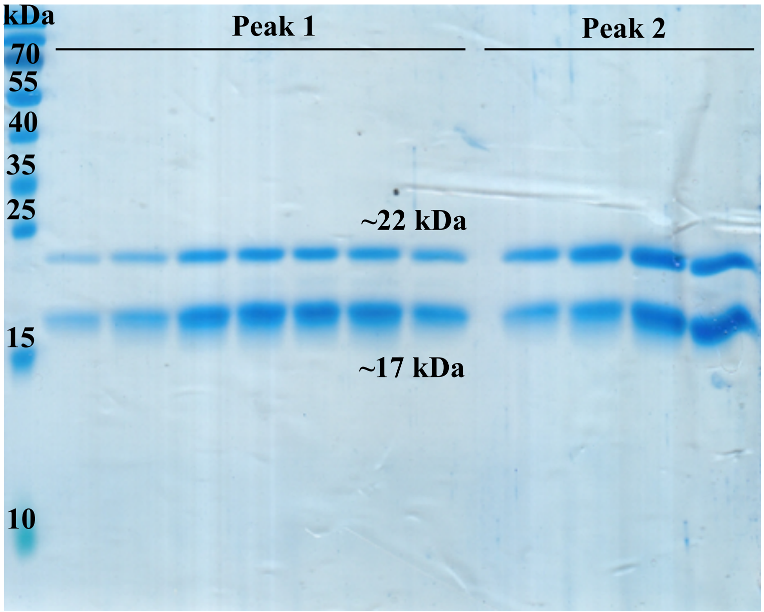

Figure S2. SDS-PAGE profile shows partial protein truncation of YuiC K32-E218. The truncation is seen in both the dimer (peak 1) and monomer (peak 2) peak. Lanes are fractions sampled across a gel filtration run.

Supplementary Figure 3

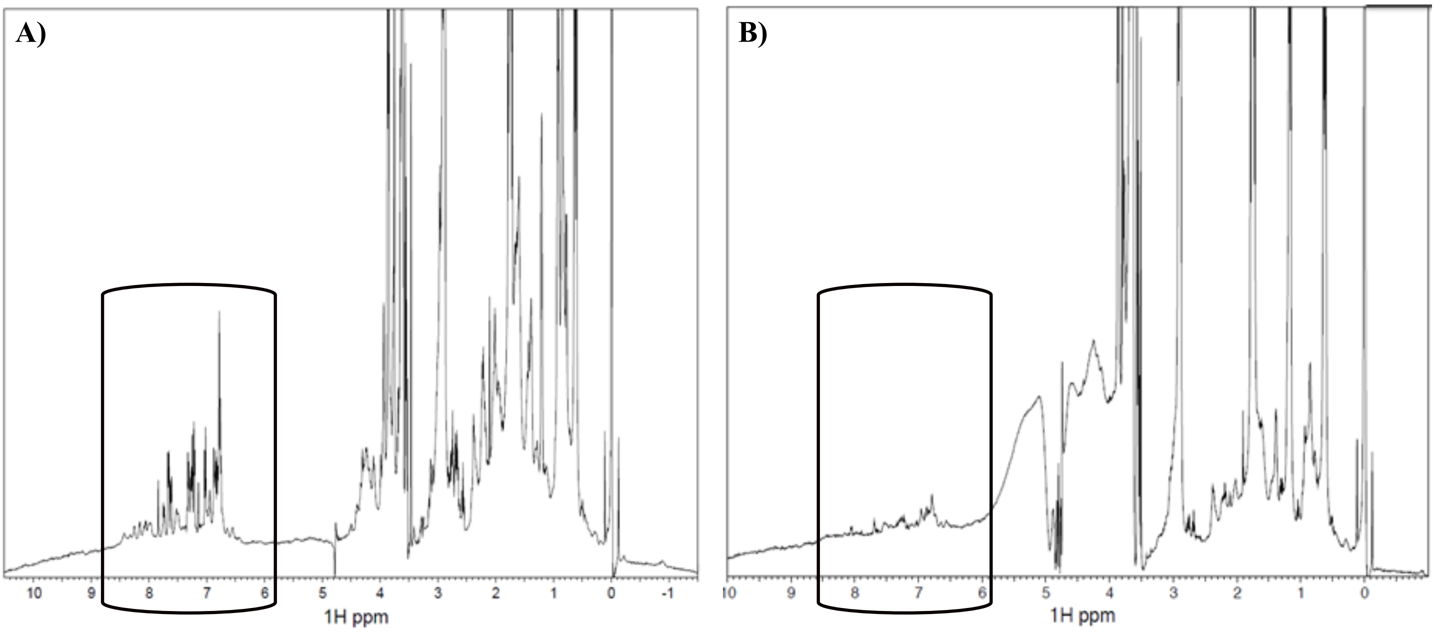

Figure S3. 1D NMR profiles of YuiC K32-E218. A) Untruncated protein sample. B) Partially truncated protein sample. The difference in the a mide region between both profiles is showed in a black framed box and the loss of some sharper peaks indicated an unfolded region has been removed . Spectra were collected at 500 μ M in 20mM Tris pH 8.0, 50mM NaCl 10% D_2O in a Shigemi tube using a 600 MHz Varian Inova Spectrophotometer.
